# Supplementary material for: Entropy-stabilized single-atom Pd catalysts via high-entropy fluorite oxide supports
Source: Nat Commun. 2020 Aug 6;11:3908. doi: 10.1038/s41467-020-17738-9 (PMC7413391; doi:10.1038/s41467-020-17738-9)
Supplement: Supplementary file 1 — Supplementary Information [file 41467_2020_17738_MOESM1_ESM.pdf]

Supplementary Information

Entropy-Stabilized Single-Atom Pd Catalysts via High-Entropy Fluorite Oxide Supports

Xu et al.

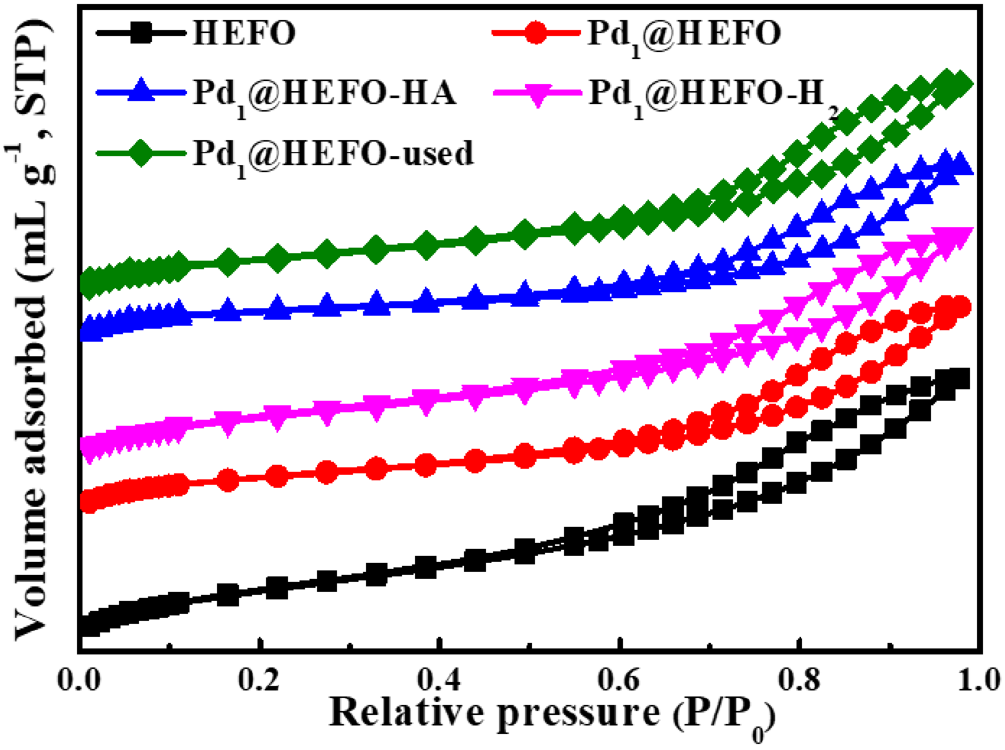

Supplementary Figure 1. **N_2 adsorption-desorption isotherms of HEFO and Pd_1@HEFO treated under different conditions. Source data are provided as a Source Data file.**

Supplementary Note 1

N_2 adsorption–desorption isotherms show that the shape of isotherm and surface area is not affected after introducing Pd and (hydro)thermal treatment.

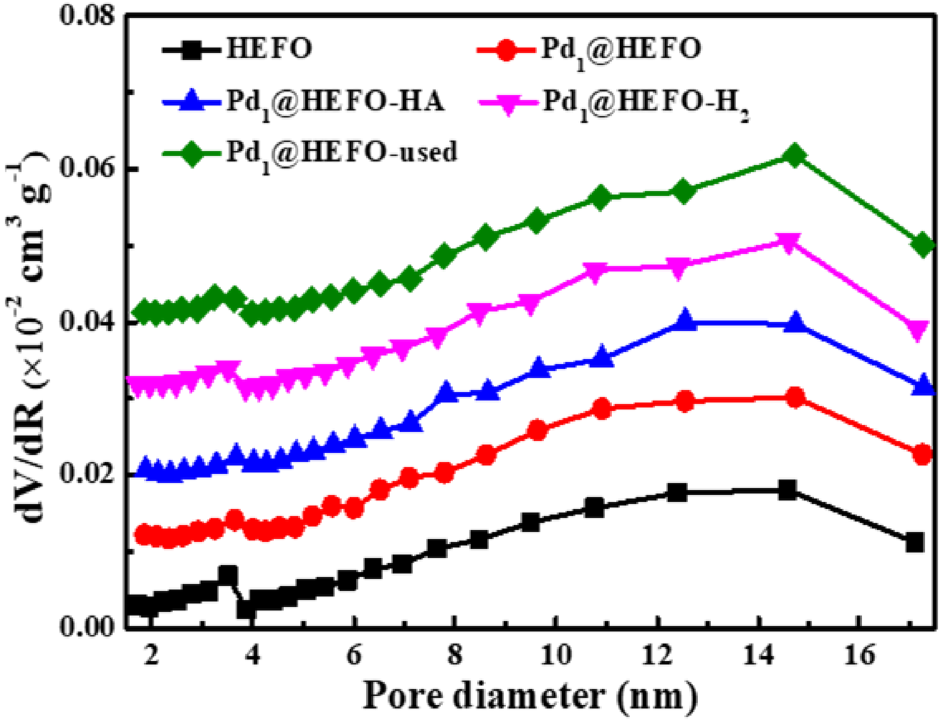

Supplementary Figure 2. Pore size distribution of HEFO and Pd_1@HEFO treated under different conditions. Source data are provided as a Source Data file.

Supplementary Note 2

Pore size distribution implies that the pore size distribution is not influenced after introducing Pd and (hydro)thermal treatment.

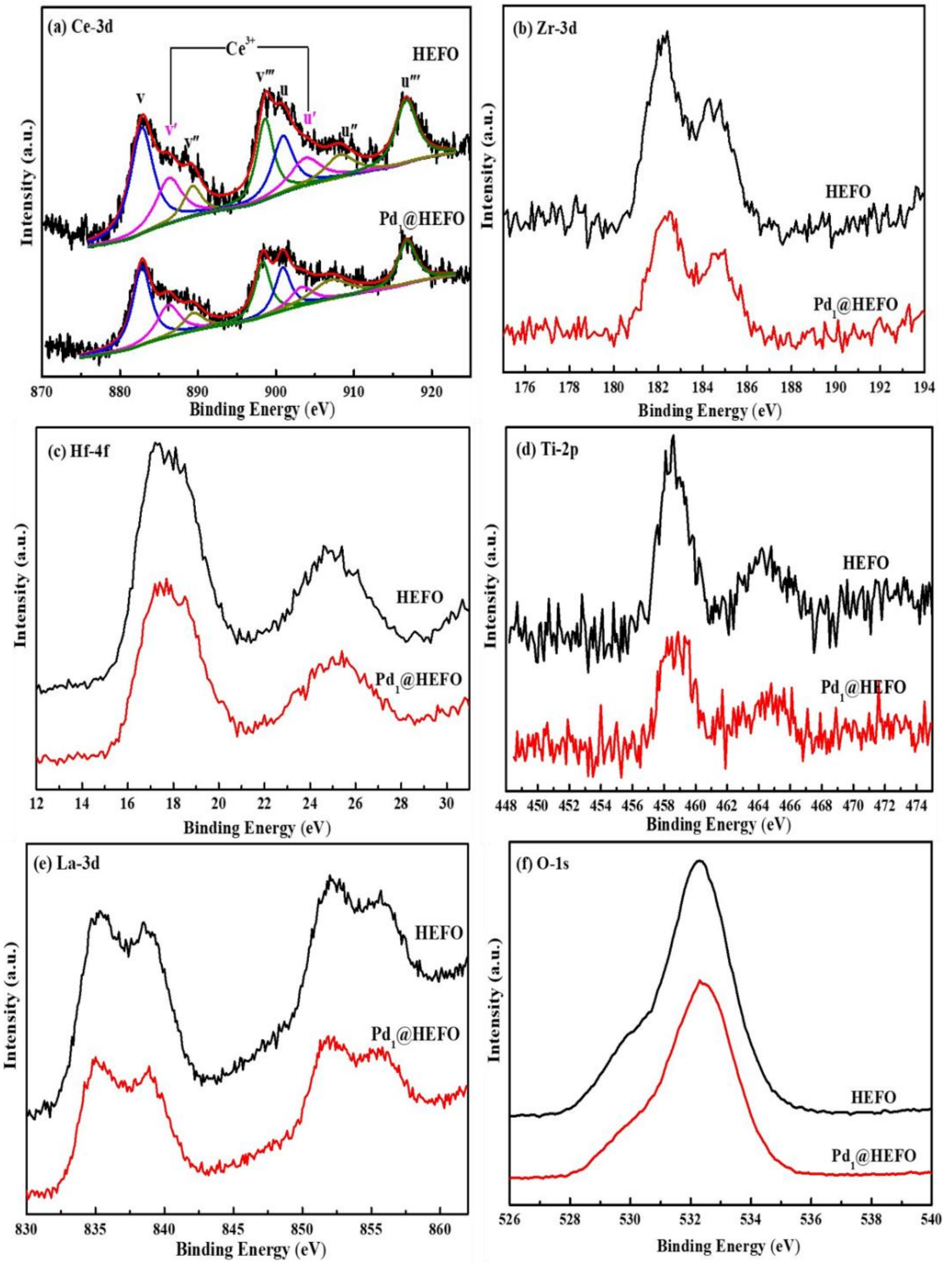

Supplementary Figure 3. **XPS profiles of (a) Ce 3*d*, (b) Zr 3*d*, (c) Hf 4*f*, (d) Ti 2*p*, (e) La 3*d*, and (h) O 1*s* for HEFO and Pd_1@HEFO. Source data are provided as a Source Data file.**

Supplementary Note 3

The surface of HEFO is mainly dominated by Zr^{4+}, Hf^{4+}, Ti^{4+}, Ce^{4+}, and La^{3+}, as elucidated by XPS, which is not influenced by the introduction of Pd into HEFO.

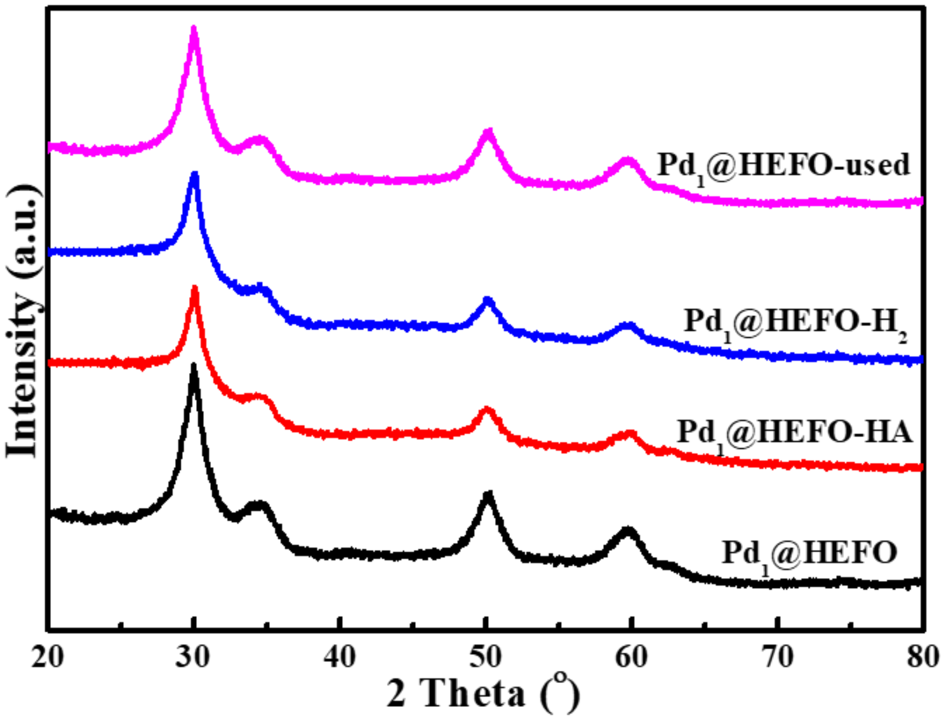

Supplementary Figure 4. **XRD patterns of Pd_1@HEFO treated under different conditions. Source data are provided as a Source Data file.**

Supplementary Note 4

The crystalline structure of Pd_1@HEFO is not influenced after (hydro)thermal treatment.

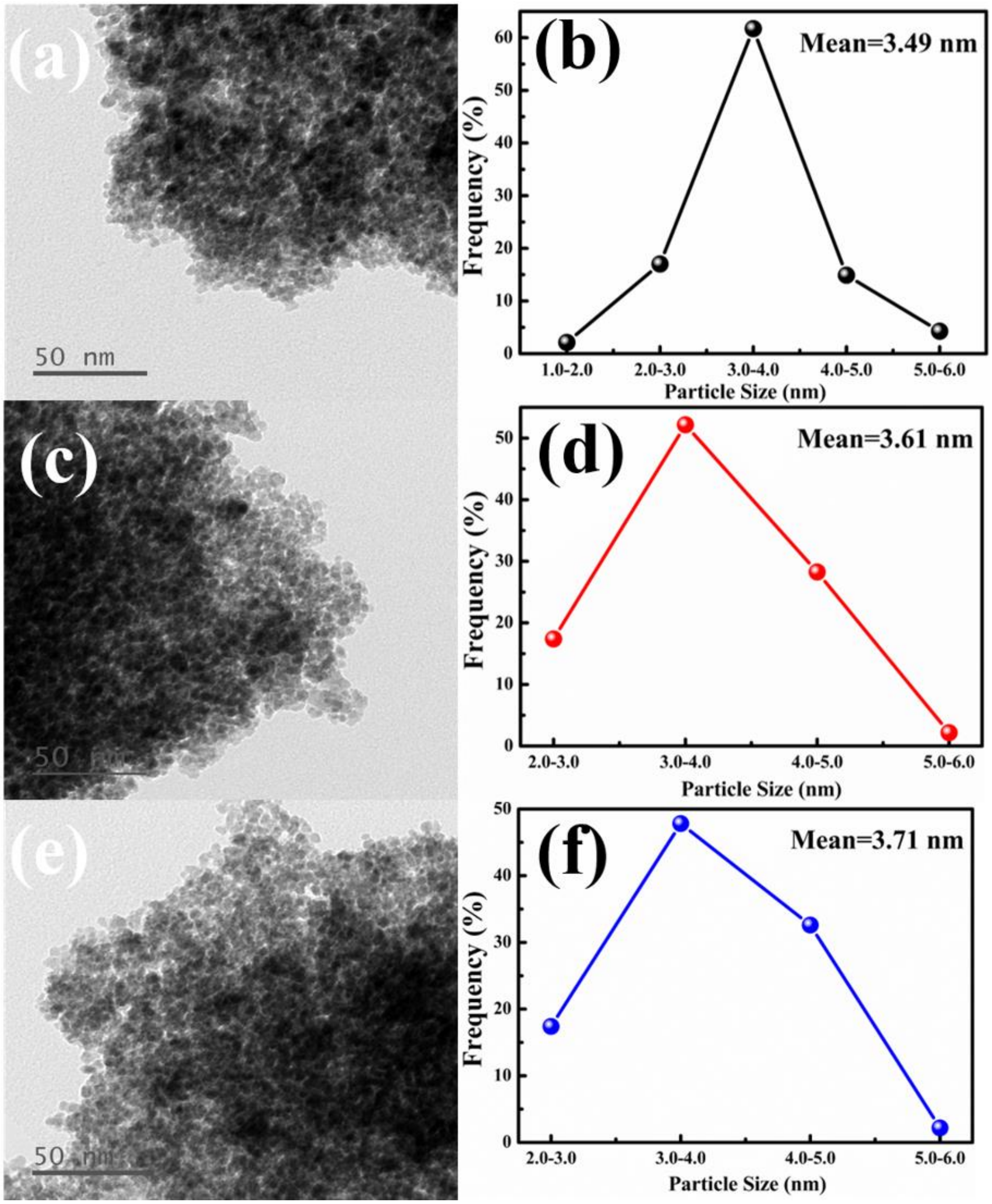

Supplementary Figure 5. TEM images and particle size distributions of (a) (b) HEFO, (c) (d) Pd_1@HEFO, and (e) (f) Pd_1@HEFO-HA. Source data are provided as a Source Data file.

Supplementary Note 5

The TEM images show that the morphology and grains size is not affected by the incorporation of Pd into HEFO and hydrothermal treatment. The grains size distribution for all is centered around 4 nm.

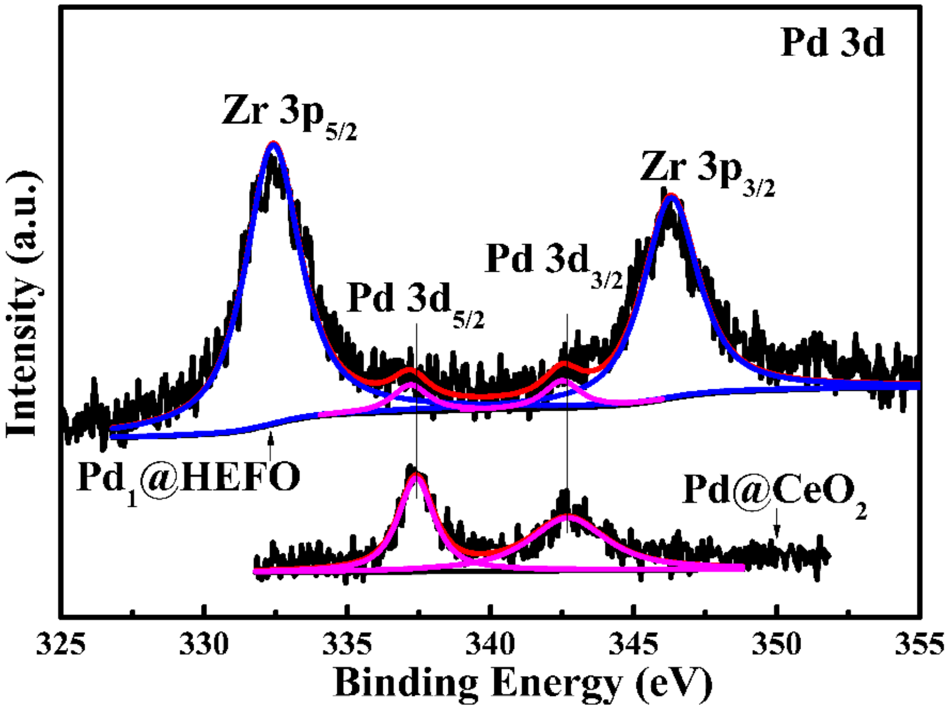

Supplementary Figure 6. Pd 3*d* and Zr 3*p* XPS spectra of Pd_1@HEFO and Pd@CeO_2. Source data are provided as a Source Data file.

Supplementary Note 6

The obtained binding energy is the characteristic of electron-deficient Pd^{4+} both in Pd_1@HEFO and Pd@CeO_2.

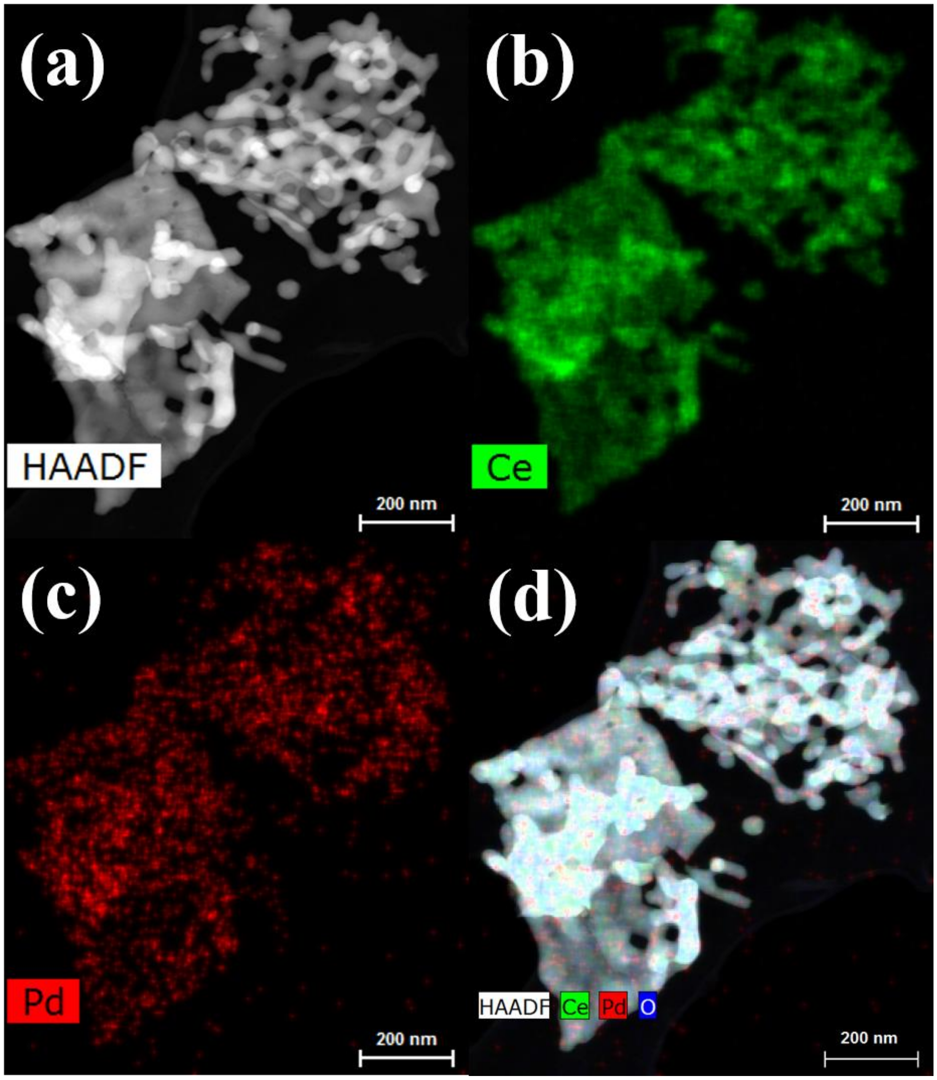

Supplementary Figure 7. **(a) EDS-mapping of (b) Ce, (c) Pd, and (d) their combined image of Pd@CeO_2.**

Supplementary Note 7

Compared with Pd_1@HEFO, obvious Pd clusters are observed from the EDS-Mapping of Pd@CeO_2 with the same synthesis of Pd_1@HEFO, demonstrating the trait of HEFO as the carrier of SACs rather than CeO_2.

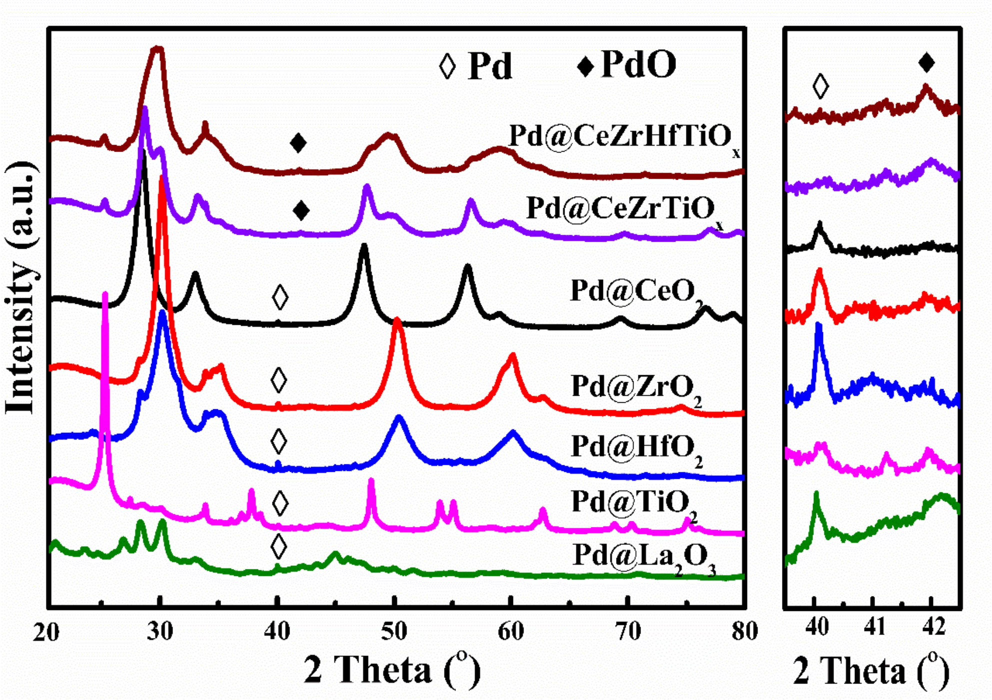

Supplementary Figure 8. PXRD pattern of Pd@CeO_2, Pd@ZrO_2, Pd@HfO_2, Pd@TiO_2, Pd@La2O_3, Pd@$CeZrTiO_x$, and Pd@$CeZrHfTiO_x$. Source data are provided as a Source Data file.

Supplementary Note 8

In order to confirm the interaction between Pd and one of the five elements, Pd@CeO_2, Pd@ZrO_2, Pd@La_2O_3, Pd@HfO_2, Pd@TiO_2, ternary Pd@$CeZrTiO_x$, and quaternary Pd@$CeZrHfTiO_x$ were synthesized via the same method of Pd_1@HEFO. From XRD patterns of all samples, a weak diffraction peak at ca. 40.20° ascribed to metallic Pd was clearly observed in all samples for Pd supported on pure metal oxides. However, its intensity in Pd@$CeZrTiO_x$ and Pd@$CeZrHfTiO_x$ was too low to detect. Another weak peak at ca. 42.00° assigned to PdO was also detected in all samples, which is expected for traditional Pd@CeO_2. However, no peaks attributes Pd or PdO_x can be observed in Pd@$CeZrHfTiLaO_x$ from Fig. 2, which illustrates that the formation of stable Pd SACs (Pd_1@HEFO) originate from the high configurational entropy lattice rather than the strong interactions between Pd and one of the five elements.

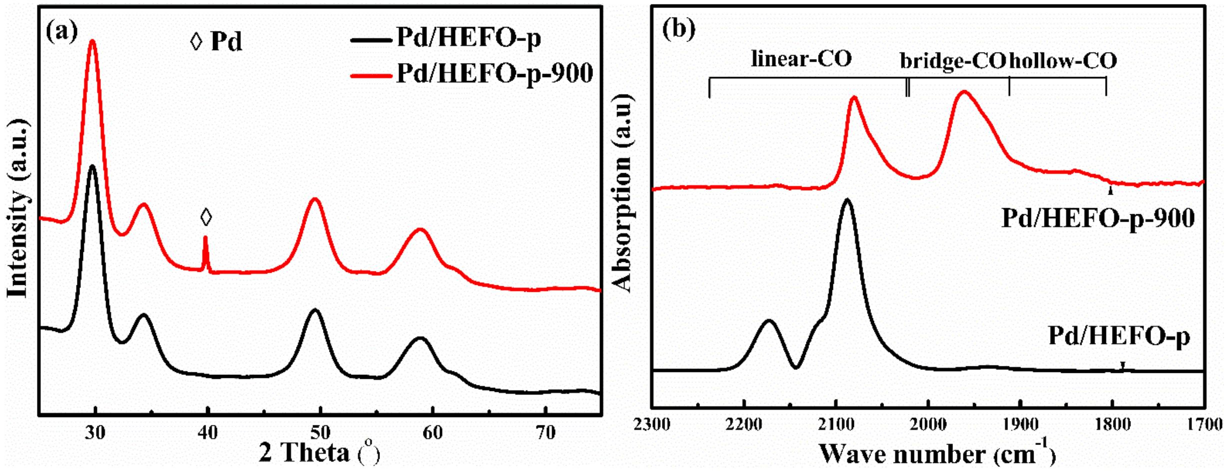

Supplementary Figure 9. (a) PXRD patterns and (b) CO-DRIFTS spectra of Pd/HEFO and Pd/HEFO-900. Source data are provided as a Source Data file.

Supplementary Note 9

No diffraction peaks attributed to Pd or PdO_x species can be observed in Pd/HEFO from Supplementary Figure 9a. Moreover, only CO frequencies between 2200 and 2000 cm^{-1} were clearly seen in Pd/HEFO-p from Supplementary Figure 9b, which is assignable to CO molecules linearly adsorbed on single-atom Pd species in Pd/HEFO-p. After calcined at 900 °C, a sharp peak at ca. 40.18° was observed in XRD pattern of Pd/HEFO-p-900 from Supplementary Figure 9a. Correspondingly, the bridge-CO and hollow-CO bands are obviously observed for Pd/HEFO-900 because of the aggregation of Pd species (Supplementary Figure 9b). The combination of both XRD and CO-DRIFTS results of Pd/HEFO-p and Pd/HEFO-p-900 indicates that Pd can be single-atom dispersed on HEFO via a facile adsorption method. However, the sintering and aggregation of Pd can be obviously observed in Pd/HEFO after calcination at 900 °C. This observation illustrated that the formation of single Pd atoms and the phase formation of high-entropy fluorite oxides proceed simultaneously during the synthesis process, because the formation of Pd−O−M bonds can stabilize single Pd atoms on HEFO for Pd_1@HEFO.

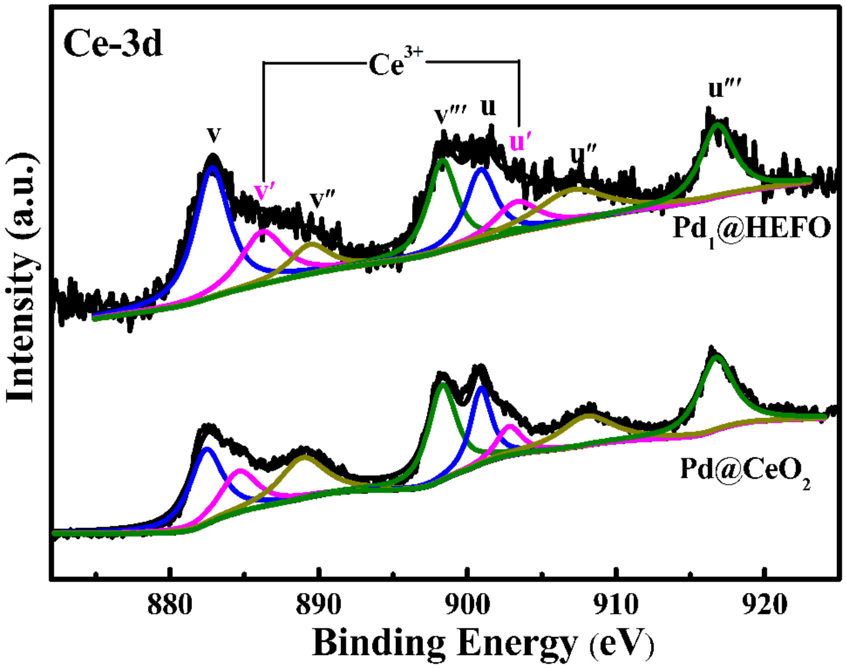

Supplementary Figure 10. XPS spectra of Ce 3*d* of Pd_1@HEFO and Pd@CeO_2. Source data are provided as a Source Data file.

Supplementary Note 10

The XPS spectra of Ce 3*d* of Pd_1@HEFO and Pd@CeO_2 are plotted in Supplementary Fig. 10. It can be seen that Ce 3*d* spectrum was composed of two serial spin orbital multiplets labelled v and u related to four different spin-orbit doublets by the Gaussian-Lorentz fitting procedure. The bands labels as v stand for $3d_{5/2}$ spin-orbit states and those labelled as u represented $3d_{3/2}$ states. Among them, the doublet v′ and u′ are ascribed to the primary photoemission of Ce^{3+}, whereas the other six bands labels as v and u, v″ and u″, v‴and u‴ were attributed to Ce^{4+}. The relative amount of Ce^{3+} could be calculated by the following formula: Ce^{3+}% =S(v′)+S(u′)/[S(v)+S(u)]∗ 100%. The ratio of Ce^{3+} in Pd_1@HEFO is 19.2% which is higher than 10.9% of Pd@CeO_2.

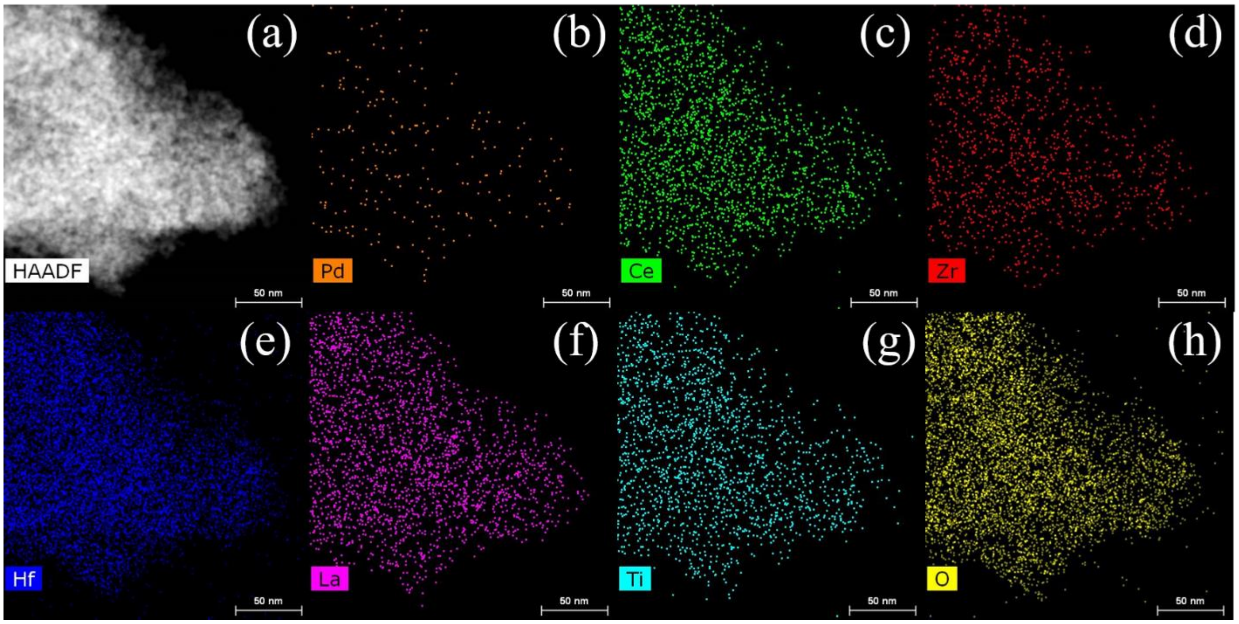

Supplementary Figure 11. (a) EDS elements mapping of (b) Pd, (c) Ce, (d) Zr, (e) Hf, (f) Ti, (g) La and (h) O, for Pd_1@HEFO-HA sample.

Supplementary Note 11

After introducing Pd into HEFO, all elements are still uniformly dispersed from EDS-mapping without agglomeration and sintering of Pd species detected in Supplementary Figure 11.

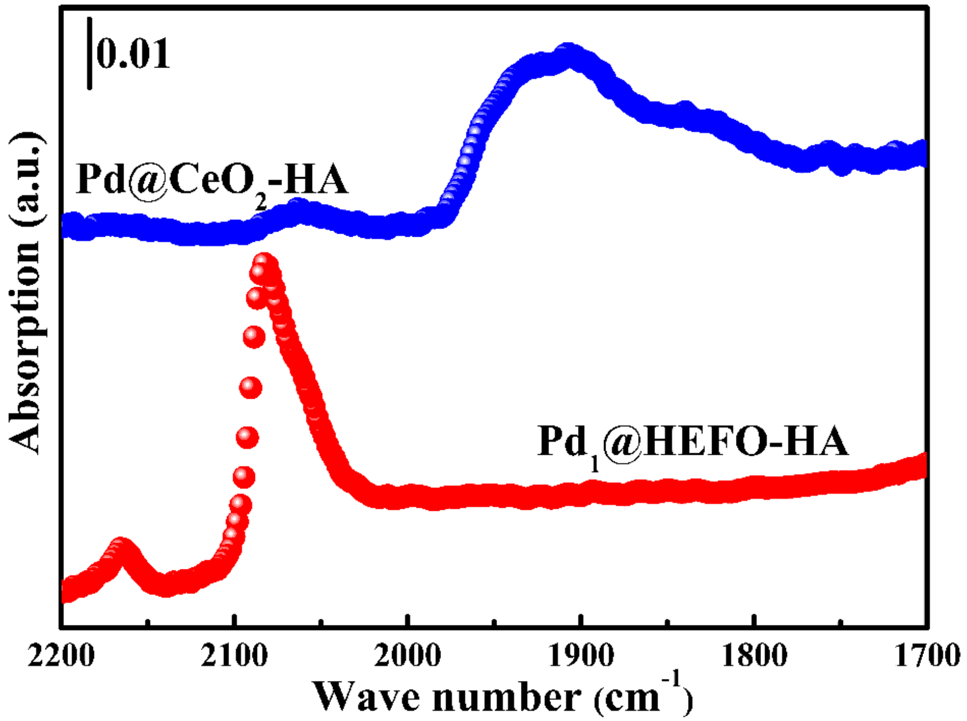

Supplementary Figure 12. CO-DRIFTS results of Pd_1@HEFO-HA and Pd/CeO_2-HA. Source data are provided as a Source Data file.

Supplementary Note 12

CO-DRIFTS results show that the absence of the bridge and hollow-CO peaks further validates the existence of single-atom Pd in Pd_1@HEFO-HA, while the weak linear-CO peak and strong bridge and hollow-CO peaks implies the presence of highly-dispersed Pd in Pd/CeO_2.

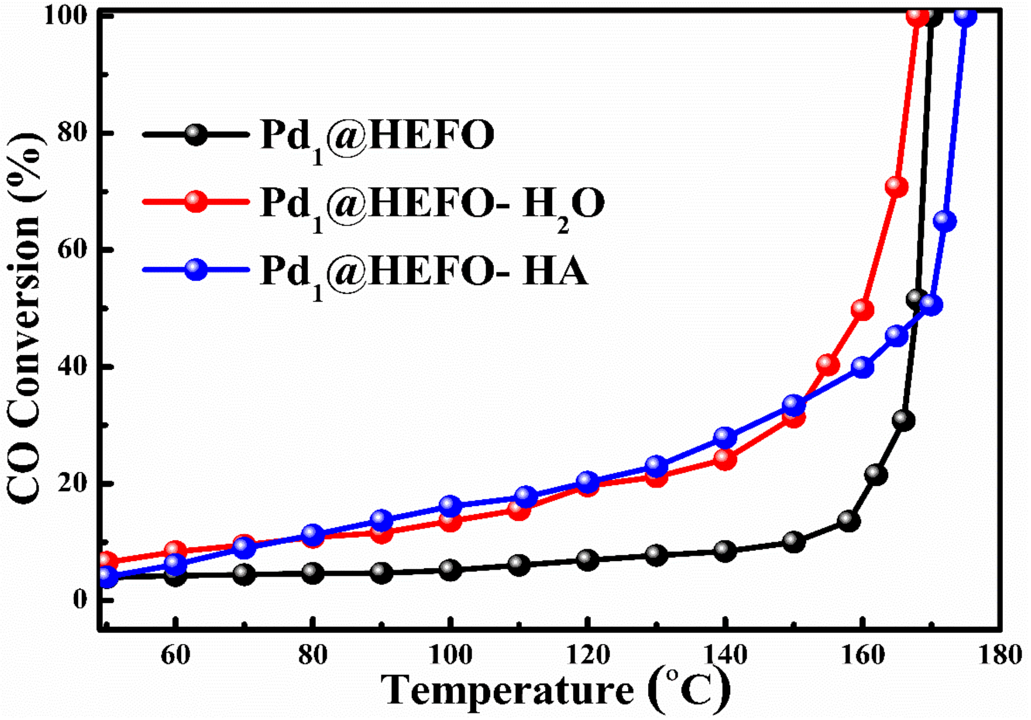

Supplementary Figure 13. **The effect of H_2O on the catalytic activity of CO oxidation over Pd_1@HEFO. Reaction conditions: A catalyst loading of 20 mg, 1 vol.% CO, and 10 vol.% H_2O balance in air at a gas hourly space velocity of 40,000 mL $gcat^{-1}$ $hour^{-1}$. Source data are provided as a Source Data file.**

Supplementary Note 13

In the presence of 10 vol.% H_2O in the feed, the catalyst shows no deactivation from Supplementary Figure 13. Instead, the activity of CO oxidation is slightly enhanced in the presence of 10 vol.% H_2O, especially in the low-temperature ranges, which is similar as that of Pd_1@HEFO-HA. It suggests that Pd_1@HEFO exhibited a good tolerance with H_2O.

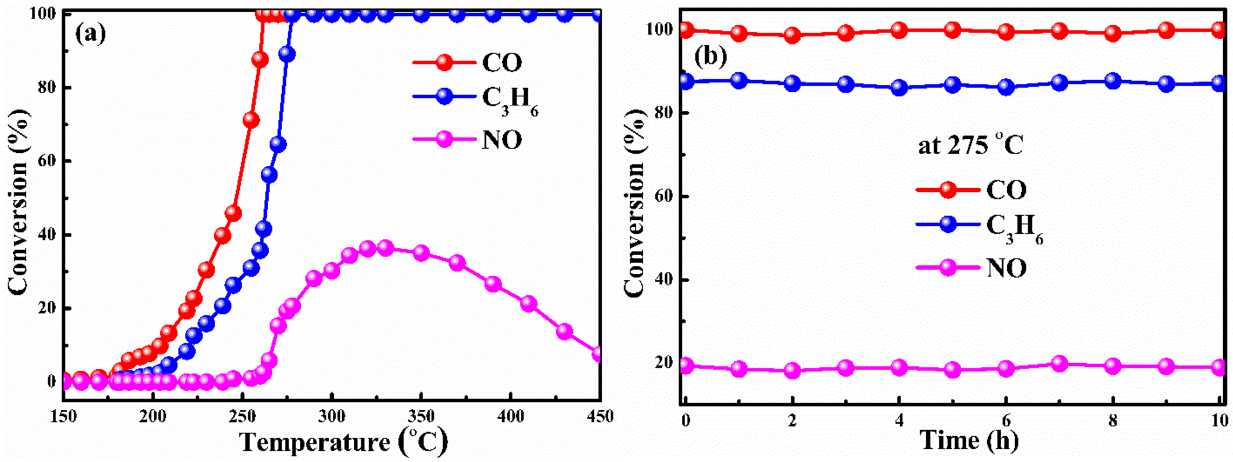

Supplementary Figure 14. (a) CO, C_3H_6, and NO oxidation activity and (b) the stability of Pd_1@HEFO. Reaction conditions: 1000 ppm CO, 330 ppm C_3H_6, 200 ppm NO, 10% O_2, N_2 balance, a catalyst loading of 150 mg at a high gas hourly space velocity of 200,000 mL $gcat^{-1}$ $hour^{-1}$ with 500 mL min^{-1} of the total gas flow rate. Source data are provided as a Source Data file.

Supplementary Note 14

CO, C_3H_6, and NO oxidation activity of Pd_1@HEFO as a diesel oxidation catalyst (DOC) are supplemented in Supplementary Figure 14a. Pd_1@HEFO exhibited excellent oxidation activities of CO, C_3H_6, and NO at a high gas hourly space velocity (GHSV) of 200,000 mL $gcat^{-1}$ $hour^{-1}$, although the T_{100} of CO oxidation was shifted to ca. 260 °C due to the co-presence of C_3H_6 and NO and an ultral-high GHSV, which can compare well with that of Pt/CeO_2-SiAlO_x as a candidate of DOC[1] and that of Pt/CeO_2[2] under DOC conditions. Moreover, no obvious deactivation of CO, C_3H_6, and NO oxidation can be observed over Pd_1@HEFO in 10 hours from Supplementary Figure 14b, suggesting that Pd_1@HEFO showed a good DOC activity and stability under a high GHSV, suggesting that entropy-stabled Pd SACs on HEFO can be a candidate of DOC for eliminating emissions from diesel engines.

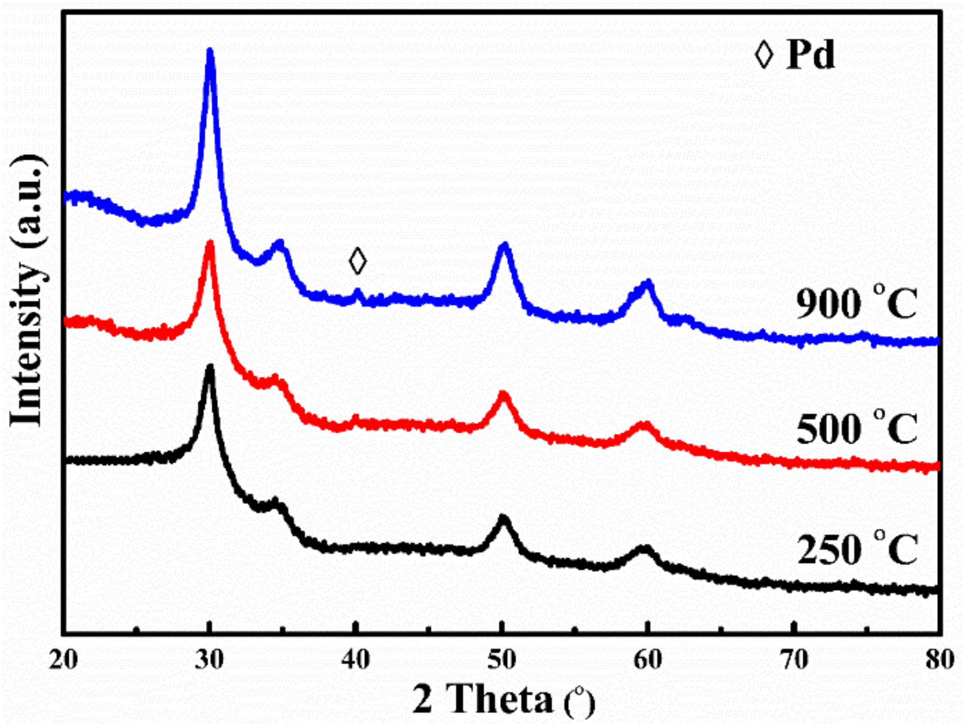

Supplementary Figure 15. XRD patterns of Pd_1@HEFO after H_2 treatment at different temperatures for 2 h. Source data are provided as a Source Data file.

Supplementary Note 15

No peaks assigned to metallic Pd or PdO_x can be observed in the sample treated at 250 °C; however, a weak peak at ca. 40.20° attributed to metallic Pd was detected when the treated temperature was increased to 500 °C. The peak intensity was slightly increased when the temperature was further increased to 900 °C. It suggested that single Pd atoms can be stabilized on HEFO in the reductive atmosphere at low temperatures (such as 250 °C). However, further increasing reductive temperature resulted in the sintering and aggregation of reduced Pd. Pd_1@HEFO calcined at 900 °C in air displayed an excellent performance of CO oxidation, illustrating that the catalyst is suitable for the oxidation reaction under oxygen-rich conditions.

Supplementary Table 1. Elemental concentrations (atom. %) in Pd_1@HEFO and Pd@CeO_2 obtained from EDS and ICP.

Samples	Methods	Pd	Ce	Zr	Hf	Ti	La	Chemisorption(μmol CO/μmol Pd)[a]
Pd_1@HEFO	EDS	0.52%	7.08%	7.39%	7.84%	7.44%	7.49%	0.0644
	ICP	0.25%	3.45%	3.49%	3.59%	3.55%	3.48%	
Pd@CeO_2	EDS	0.53%	35.69%	-	-	-	-	-
	ICP	0.24%	14.87%	-	-	-	-	

[a] CO chemisorption measurements were carried out on a Micromeritics AutoChem II 2920.

Supplementary Note 16

The results of EDS and ICP both show that the molar ratio of Ce:Zr:Hf:Ti:La is close to 1:1:1:1:1 for Pd_1@HEFO, further demonstrating that the formation of equimolar high-entropy fluorite oxides. Moreover, the atom concentrations of Pd in both samples are similar as seen from both EDS and ICP.

Supplementary Table 2. Textural properties of HEFO, Pd_1@HEFO with different treated conditions and Pd_1@HEFO-*x* with different Pd loading.

Sample	BET Surface area (m^2/g)	Pore volume (cm^3/g)	Pore size (nm)
HEFO (900 °C)	162.1	0.2446	6.04
Pd_1@HEFO-1.0	136.5	0.2099	6.15
Pd_1@HEFO-HA	109.6	0.1695	6.19
Pd_1@HEFO-H_2	127.8	0.1975	6.18
Pd_1@HEFO-used	131.8	0.2037	6.18
Pd_1@HEFO-0.5	157.4	0.2258	5.73
Pd_1@HEFO-1.5	119.1	0.1839	5.84
Pd_1@HEFO-2.0	117.3	0.1653	5.64

Supplementary Note 17

Supplementary Table 2 shows that HEFO as the carrier possesses a high surface area for single-atom dispersion. The incorporation of Pd slightly influences the textural properties of HEFO, and the surface area of Pd_1@HEFO-*x* increasingly declined by enhancing the loading of Pd until 1.5 wt.%. Importantly, Pd_1@HEFO displays a strong stability because the surface area is not affected by different thermally treating conditions.

Supplementary Table 3. Best fitting parameters obtained from Pd K-edge EXAFS analysis of Pd_1@HEFO, PdO and Pd foil. (S_0^2=0.829).

Sample	Shell	CN^a	R(Å)^b	σ^2(Å^2)^c	ΔE_0(eV)^d	R factor
Pd_1@HEFO	Pd−O	4.3	2.01	0.0062	-0.9	0.0151
	Pd−O−Zr	3.7	3.01	0.0086		
	Pd−O−M^e	5.0	3.26	0.0086		
PdO	Pd−O	4.0	2.02	0.0027	-0.9	0.0049
	Pd−O−Pd	4.1	3.06	0.0043		
	Pd−O−Pd	7.5	3.44	0.0043		
	Pd−O	7.3	3.68	0.0062		
Pd foil	Pd−Pd	12	2.74	0.0056	3.6	0.0013

[a]CN: coordination numbers; [b]R: bond distance; [c]σ^2: Debye-Waller factors; [d] ΔE_0: the inner potential correction. R factor: goodness of fit. S_0^2 was set to 0.829, according to the experimental EXAFS fit of Pd foil reference by fixing CN as the known crystallographic value. [e] M: M = Ce/La.

Supplementary Note 18

Pd K-edge EXAFS analysis exhibits that Pd incorporates into the lattice of HEFO and form the single-atom Pd due to the formation of Pd−O−Zr and Pd−O−M (M= Ce/La).

Supplementary References

1. Ding, X., Qiu, J., Liang, Y., Zhao, M., Wang. J. & Chen, Y. New insights into excellent catalytic performance of the Ce-modified catalyst for CO oxidation, *Ind. Eng. Chem. Res.* **58**, 7876−7885 (2019).

2. Jones, J. *et al*. Thermally stable single-atom platinum-on-ceria catalysts via atom trapping, *Science* **353**, 150–154 (2016).
